# ST-segment elevation myocardial infarction due to a giant coronary artery aneurysm caused by IgG4-related disease

**DOI:** 10.1093/ehjimp/qyae011

**Published:** 2024-02-12

**Authors:** Yasuhiro Honda, Kensaku Nishihira, Mitsuhiro Yano, Atsuko Yokota, Yujiro Asada

**Affiliations:** Department of Cardiology, Miyazaki Medical Association Hospital, 1173 Arita, Miyazaki 880-2102, Japan; Department of Cardiology, Miyazaki Medical Association Hospital, 1173 Arita, Miyazaki 880-2102, Japan; Department of Cardiovascular Surgery, Miyazaki Medical Association Hospital, Miyazaki, Japan; Department of Cardiovascular Surgery, Miyazaki Medical Association Hospital, Miyazaki, Japan; Department of Pathology, Miyazaki Medical Association Hospital, Miyazaki, Japan

**Keywords:** coronary aneurysm, histopathology, immunoglobulin G4‐related disease, ST‐segment elevation myocardial infarction

A 78-year-old male presented to the emergency department with intermittent chest pain that had worsened over the past week. Electrocardiography showed significant ST-segment elevation in leads V2–V5 (*Panel A*). Echocardiography revealed severe hypokinesia in the left anterior descending artery (LAD) territory and circumferential pericardial effusion. Laboratory testing showed a markedly elevated troponin I level (5.409 ng/mL, normal reference value: 0.032 ng/mL). Emergent coronary angiography demonstrated a giant coronary aneurysm in the proximal portion of the LAD, with occlusion in the distal portion of the aneurysm (*Panels B1* and *B2*; see Supplementary data online, *Video S1*). Since the lesion was unsuitable for percutaneous coronary intervention and there was a possibility of impending rupture of the aneurysm, coronary aneurysm resection and coronary artery bypass grafting were performed. Pre-operative computed tomography showed a giant coronary aneurysm (67 mm) (*Panels C1–C3*, arrows), significant wall thickening in the right coronary artery (*Panel C4*, arrowhead), and pericardial effusion (*Panel C3*, asterisks). Histopathological examination of the resected coronary aneurysm showed numerous IgG4-positive plasma cells with fibrosis (*Panels D1–D4*). Serum IgG4 level was highly elevated at 3062 mg/dL (normal reference value: 121 mg/mL). Thus, the patient was diagnosed with ST-segment elevation myocardial infarction (STEMI) due to a giant coronary artery aneurysm caused by IgG4-related disease.

**Figure:**
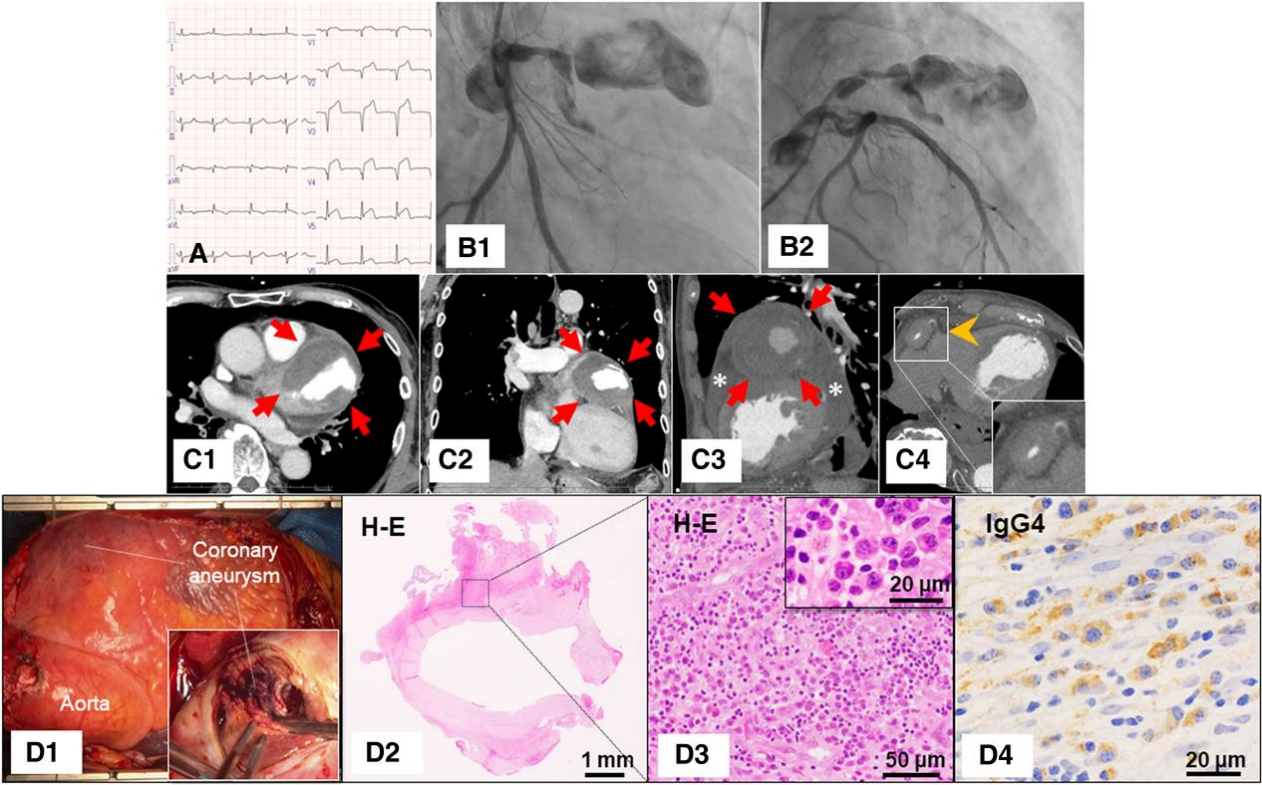


Coronary artery involvement is rare in IgG4-related disease, especially the development of STEMI. Our findings highlight the importance of considering IgG4-related disease as a potential cause of coronary aneurysms and the need for multidisciplinary management.

## Supplementary Material

qyae011_Supplementary_Data

## Data Availability

No new data were generated or analysed in support of this article.

## Supplementary data

**Consent:** The authors confirm that written consent for the submission and publication of this case report, including images and associated text, has been obtained from the patient.

**Funding:** None.

## Lead author biography

Dr Yasuhiro Honda graduated from Faculty of Medicine, Hiroshima University, in 2013. Currently, he works as an interventional cardiologist at Miyazaki Medical Association Hospital. He spends most of his work on percutaneous coronary intervention and structural heart diseases.

